# Introduction to the special issue: challenges and opportunities in the fight against neglected tropical diseases: a decade from the London Declaration on NTDs

**DOI:** 10.1098/rstb.2022.0272

**Published:** 2023-10-09

**Authors:** Kathryn Forbes, Maria-Gloria Basáñez, T. Déirdre Hollingsworth, Roy M. Anderson

**Affiliations:** ^1^ London Centre for Neglected Tropical Disease Research (LCNTDR), Department of Infectious Disease Epidemiology, School of Public Health, Faculty of Medicine, Imperial College London, London W2 1PG, UK; ^2^ MRC Centre for Global Infectious Disease Analysis (MRC GIDA), Department of Infectious Disease Epidemiology, School of Public Health, Faculty of Medicine, Imperial College London, London W2 1PG, UK; ^3^ Big Data Institute, Nuffield Department of Medicine, Oxford University, Oxford OX3 7LF, UK

**Keywords:** neglected tropical diseases, epidemiology, control, elimination, eradication, World Health Organization

## Abstract

Twenty neglected tropical diseases (NTDs) are currently prioritised by the World Health Organization for eradication, elimination as a public health problem, elimination of transmission or control by 2030. This issue celebrates progress made since the 2012 London Declaration on NTDs and discusses challenges currently faced to achieve these goals. It comprises 14 contributions spanning NTDs tackled by intensified disease management to those addressed by preventive chemotherapy. Although COVID-19 negatively affected NTD programmes, it also served to spur new multisectoral approaches to strengthen school-based health systems. The issue highlights the needs to improve impact survey design, evaluate new diagnostics, understand the consequences of heterogeneous prevalence and human movement, the potential impact of alternative treatment strategies and the importance of zoonotic transmission.

This article is part of the theme issue ‘Challenges and opportunities in the fight against neglected tropical diseases: a decade from the London Declaration on NTDs’.

## 1. The neglected tropical diseases (NTDs) and the World Health Organization roadmaps on NTDs

The neglected tropical diseases (NTDs) are a diverse group of, mainly infectious diseases (including viral, bacterial, protozoan and helminth infections) that mostly affect impoverished communities, with a disproportionate impact on women and children. They prevail in tropical and subtropical conditions of the world, and 1.65 billion people are estimated to be infected with at least one NTD worldwide. Currently, the World Health Organization (WHO) prioritises 20 NTDs [1,2]. The WHO Africa region accounts for over 35% (about 400 million people) of the global NTD burden [2,3]. While NTDs are rarely considered fatal (with the exception of Chagas disease, human African trypanosomiasis and visceral leishmaniasis), they have a devastating impact on the health, quality of life and socio-economic development of entire communities. The epidemiology of NTDs is often complex, with lifecycles that typically involve the environment, vectors, zoonotic reservoirs, intermediate and definitive hosts, making their control challenging. The commitment made in 2012, by pharmaceutical companies, donors, endemic countries and non-governmental organizations to control, eradicate or eliminate 10 NTDs by 2020 in the London Declaration on NTDs, was a key turning point in the fight against NTDs [4]. The Declaration was inspired by the first WHO roadmap on NTDs [5], and since 2012 much progress has been made.

In 2019, the WHO embarked on a consultation with the broader NTD community to revise the goals previously proposed for the 2012–2020 roadmap [5]. This was to review, in light of experience and past impact of control activities, the technical feasibility of achieving the targets with current interventions, and to identify challenges ahead, new tools that may be necessary and important risks to be mitigated in the next 10 years. The outcomes of this process were published in a new roadmap: ‘Ending the neglect to attain the Sustainable Development Goals: A road map for neglected tropical diseases 2021–2030’ [6]. This revised roadmap identifies NTDs for which the target is eradication (e.g. yaws; dracunculiasis (Guinea worm)); elimination (interruption) of transmission (EOT; e.g. infections not deemed zoonotic, such as onchocerciasis and (gambiense) human African trypanosomiasis; gHAT); elimination as a public health problem (EPHP; e.g. lymphatic filariasis (LF); schistosomiasis), and control (e.g. zoonotic infections such as *Taenia solium* taeniasis/cysticercosis and hydatid disease). Table 1 provides a list of the 20 NTDs currently considered by the WHO and their 2030 goals. The roadmap also defined interim goals for 2023–2025 [6].

**Table 1.** The 20 neglected tropical diseases (NTDs)^a^ currently prioritized by the World Health Organization (WHO) and their 2030 targets [6].

eradication	elimination (interruption) of transmission (EOT)
1. yaws	11. leprosy
2. dracunculiasis (Guinea worm)	6b. (gambiense) human African trypanosomiasis (gHAT)
	12. onchocerciasis
**elimination as a public health problem (EPHP)**	**control**
3. rabies	13. dengue
4. trachoma	14. Buruli ulcer
5. American trypanosomiasis (Chagas disease)	15. mycetoma, chromoblastomycosis and other deep mycoses
6a. (rhodesiense) human African trypanosomiasis	7b. cutaneous leishmaniasis
7a. visceral leishmaniasis (VL)	16. echinococcosis
8. schistosomiasis	17. *Taenia solium* taeniasis/cysticercosis
9. soil-transmitted helminthiases (STH)	18. foodborne trematodiases
10. lymphatic filariasis (LF)	19. scabies and other ectoparasitoses
	20. snakebite envenoming

^a^The NTDs have been ordered according to causative agent: viruses (rabies; dengue); bacteria (yaws; trachoma; leprosy; Buruli ulcer); fungi (mycetoma; chromoblastomycosis); protozoa (Chagas disease; HAT; leishmaniasis); cestodes (echinococcosis; taeniasis); trematodes (schistosomiasis; food-borne trematodiases); nematodes (Guinea worm; STH; LF; onchocerciasis); ectoparasites (scabies); non-infectious (snakebite). For definitions of eradication, elimination and control see [7].

This timely collection of papers marks 10 years since the London Declaration and the first WHO roadmap on NTDs and celebrates the progress made over the last decade. The first paper traces the evolution of the NTD concept, its formal emergence and adoption in the year 2000, and the impetus that ensued to establish a new WHO department for NTD control in 2005 [8].

The collection goes on to discuss the challenges and opportunities that lie ahead in the journey to achieve the revised goals set in the second WHO roadmap. Three pillars provide the foundations to this roadmap: (i) accelerate programmatic action; (ii) intensify cross-cutting approaches, and (iii) facilitate country ownership. This volume brings examples of studies that can contribute to strengthening these pillars, such as the modelling analysis on the use of moxidectin mass drug administration (MDA) to accelerate onchocerciasis EOT based on clinical trial data generated by country actors [9], and multi-sectoral and country-ownership approaches for school-based deworming programmes [8].

The most recent WHO report on NTDs celebrates the vast gains that have been made, with more than one billion people treated for NTDs every year between 2016 and 2019. However, it also highlights the challenges for health systems which have arisen owing to the COVID-19 pandemic, changes in the funding landscape, and the unequal progress on NTDs, with some countries or populations being left behind, often owing to poverty, climate change and lack of infrastructure [2].

One encouraging trend towards the change in operating models and culture to facilitate country ownership that constitutes the third pillar of the WHO roadmap is the funding of country-wide control programmes by philanthropic organizations, which support much of the NTD control programmes in resource-constrained countries. The purpose is to increase support for local implementers with the aim of strengthening local capacity in NTD control. The ultimate goal is to gradually encourage local Ministries of Health to take over the NTD control programmes and support them within their own budgets, reducing dependency from foreign assistance, which up until now has been a major catalytic force [10]. As this process builds in momentum, it will be important to ensure that independent and rigorous monitoring of logistics and performance is conducted such that the gains achieved in lowering infection levels over the past decade are sustained and further built upon, with the eventual long-term goal of disease elimination [7]. Some of the most pressing issues in global health currently, such as the drive for Universal Health Coverage, the climate change crisis, and better detection of new and emerging health emergencies, can be addressed by using the power, reach and know-how of those who plan and deliver NTD programmes [11].

To this end, it is essential that NTD surveys used to measure progress are optimally designed. Current WHO guidelines set prevalence thresholds below which EPHP may have been achieved, and specify how surveys to assess this target should be designed and analysed, based on classical survey sampling methods. The paper by Diggle *et al*. argues that an alternative approach, based on geospatial statistical modelling, can lead to efficiency gains by exploiting spatial correlation in the underlying prevalence, and advocates for the use of a predictive inferential framework which naturally accommodates context-specific information in the form of georeferenced covariates that have been shown to be predictive of disease prevalence. They exemplify this approach by reporting on ongoing collaborative work with the Guyana Ministry of Health NTD programme for the design of an ivermectin + diethylcarbamazine + albendazole impact survey of LF [12].

## 2. Intensified disease management and preventive chemotherapy neglected tropical diseases

The strategies to combat NTDs are broadly categorized into two groups, those which are tackled by innovative and intensified disease management (IDM), and those that rely on preventive chemotherapy (PC) [13,14]. For the former, the primary method of control is through detection and (sometimes prolonged) treatment and management of identified cases, which requires strengthening of health systems. For the latter, PC is a cornerstone of the global NTD programme, where PC is defined as large-scale delivery of safe, quality-assured medicines, either alone or in combination, at regular intervals to entire population groups at risk, without the need for individual diagnosis before each treatment round. MDA is an important plank of PC measures for many of the parasitic infections. For example, WHO recommends PC against five NTDs, namely, LF, onchocerciasis, schistosomiasis, soil-transmitted helminthiases (STH) and trachoma (where PC with azithromycin represents the ‘A’ component of the SAFE strategy: Surgery, Antibiotics, Facial cleanliness and Environmental improvement). PC has also been used for other NTDs, including food-borne trematodiases, taeniasis and yaws (a bacterial IDM NTD), although on a smaller scale for these groups of infections. Other interventions, such as case management, vector control, veterinary public health and water, sanitation and hygiene (WASH), may also be required for the control, elimination and eradication of the burden of these NTDs [15]. Although the development and introduction of new medicines (with shorter treatment courses) and novel diagnostic tools has been prioritized and invested upon for the IDM NTDs, this aspect of research and development is equally important for the PC NTDs. For all the NTDs, the great strides that have been made have unmasked underlying heterogeneities, such as local hotspots of transmission, or populations not accessing treatments, which will need to be addressed to reach the ambitious goals for the global programme. Addressing these heterogeneities requires a better understanding of the underlying epidemiology and necessitates new tools to address them.

In fact, an issue linked to sustaining the successes gained in the fight against the PC NTDs is the need for better and more sensitive point-of-care diagnostics to ensure that pockets of low-intensity infections can be detected. Exciting opportunities exist in research laboratories based on parasite secretions detectable in blood, urine or faeces. However, the challenge is to convert these technical advances into low-cost diagnostics suitable for use in resource-constrained settings. The contribution of Kabbas-Piñango and co-authors discusses the importance of reproducibility and intra- and inter-sample variation of the point-of-care circulating cathodic antigen (POC-CCA) test in high and low *Schistosoma mansoni* endemic areas in Uganda [16], and the paper by Rotejanaprasert *et al*. presents an evaluation of Kato-Katz and multiplex qPCR performance for the diagnosis of helminth infections (food-borne trematodiasis caused by *Opisthorchis viverrini*; cestode infections caused by *Taenia solium*, and STH due to *Ascaris lumbricoides*, *Trichuris trichiura* and hookworm) in Thailand, using a Latent Class Analysis [17].

For IDM diseases, such as leprosy (targeted for EOT in the second WHO roadmap), there may be a long delay between infection and detection, and therefore, evaluating the relationship between observed cases and underlying transmission can be challenging. Due to long incubation periods, it may take many years for the final cases to be observed, making it difficult to assess progress towards elimination goals in the interim period. Davis and colleagues [18] provide an analysis of the recently published WHO technical guidance on the interruption of transmission and elimination of leprosy disease [19] in low-incidence settings based on modelling incubation period and detection delay distributions. They discuss the feasibility and challenges of such an approach to assess programme status under scenarios of transmission decline to zero incidence or low-level persistent transmission [20].

## 3. Mass drug administration

In 2021, 1.65 billion people were reported to require mass or individual treatment and care for NTDs, down from 2.19 billion in 2010, and about 80 million fewer than reported in 2020. Treatment and care is broadly defined to include preventive, curative, surgical or rehabilitative interventions. These figures include the average annual number of people requiring MDA for at least one PC-NTD; and the number of new cases requiring individual treatment and care for other NTDs [21]. Progress in NTD control has often been most significant when PC is the main template of intervention activity, and impressive successes have been recorded in reducing prevalence of PC NTD infections. However, after years of MDA, a stage has now been reached in which prevalence is low in some areas, but consistently delivering appropriately high levels of MDA across endemic and cross-border regions remains challenging. For example, a recommended WHO target for the STH and schistosomiasis is to achieve a goal of 75% coverage for school-aged children (SAC). In many countries or regions with endemic areas within countries this has proved difficult to achieve. In addition, in areas of very high transmission (with large values of the basic reproduction number, *R*_0_) or where infection is most prevalent in adults, as is the case for hookworm, the target coverage is too low to interrupt transmission [22] and may not even serve to control the heavy-intensity infections that are associated with the greatest morbidity. A similar issue arises for schistosomiasis when infection burden in adults is high [23].

Although the name STH is used as an umbrella term to include a group of intestinal nematode infections transmitted by exposure to soil contaminated with parasite eggs or larvae, the component species (*Ascaris*, *Trichuris*, hookworm, and in the 2021–2030 WHO roadmap also *Strongyloides* [6]) can have different age-infection profiles and are differently impacted by albendazole/mebendazole. Whipworm (*Trichuris*) exhibits generally lower egg reduction rates following benzimidazole treatment, *Strongyloides* is not greatly affected by it (requiring ivermectin), and the prevalence of hookworm (*Necator*, *Ancylostoma*) increases with age rather than peaking in childhood [22,24].

Bundy and colleagues open this collection with a two-part paper [8]. This begins with a brief history of the scientific and policy foundations of school-based deworming for STH (one of the largest public health interventions in low- and middle-income countries (LMIC)), and moves on to examine the impact, on children's health, of almost universal school closures in the spring of 2020 owing to the COVID-19 pandemic. This (un)natural opportunity allowed an unintended experiment in the counterfactual scenario to understand the impact on children's health when schools are closed for a prolonged time. MDA campaigns were the second most affected public health intervention globally owing to COVID-19, after mental health services [8]. The impact of school closures on young people has resulted in national governments in LMIC strengthening their resolve in using schools to deliver public health campaigns, recognizing the value they had once they were, regrettably, temporarily suspended.

Another paper directly related to MDA campaigns for STH and schistosomiasis and their broader impact is that by Bartlett and colleagues [25]. They report on a knowledge, attitudes and practices (KAP) study conducted among SAC in regions of Angola, in schools that had either received: (i) MDA for STH and WASH interventions; (ii) MDA alone; or (iii) neither. These authors concluded that the provision of school-based MDA and WASH interventions improves health behaviours at school and at home that would support reduced STH and schistosome infection in SAC. This paper contributes to the sparse body of evidence on the KAP of SAC, despite the fact that this is generally the targeted demographic for PC interventions against STH and schistosomiasis. Interestingly, the authors report improved water use and health behaviours in the homes of SAC attending schools where PC and WASH are in place, compared to those at schools with no expanded WASH intervention, and even a difference between children at schools receiving PC and no WASH compared to children at schools with no provision of either [25]. This supports the delivery of messaging given during MDA campaigns about the importance of sanitation and hygiene to prevent re-infection.

Chandrasena and colleagues discuss the challenges of eliminating remaining LF and STH pockets of infection in Sri Lanka [26], a country which has made great strides towards EPHP for both infections, but is battling to eliminate residual transmission in some key areas. Regarding LF, they propose reasons for this to be multifactorial, including areas of high endemicity requiring more rounds of MDA than routinely given, low adherence rates to the anthelmintic drugs, and environmental, climatic and physiographic conditions supporting transmission. Regarding STH, it is proposed that sanitation and hygiene habits, limited health-seeking behaviour and differential susceptibility of each species included in this group to the benzimidazoles used for MDA are potential reasons for remaining STH infections [26].

More generally, even in regions where geographical and therapeutic coverage of PC-based interventions is on average good, much heterogeneity exists within households and between villages in adherence to the treatment offered at repeated rounds of MDA. As such, even in high average coverage regions, pockets of high infection may thus persist with the implications that they can re-seed infection once PC ceases. The contribution of Collyer and collaborators to this collection highlights the importance of considering human movement in STH models to explain declines in prevalence owing to MDA and bounce-back of hookworm infection using data from the TUMIKIA trial in Kenya [27].

The paper by Kura and colleagues investigates the potential of moxidectin MDA (a drug hailing from the field of veterinary medicine but recently approved for treatment of human onchocerciasis) compared to ivermectin for accelerating onchocerciasis EOT in African settings of varying endemicity, coverage, adherence and frequency [9].

## 4. Infection and morbidity

Although it is considered that infection intensity is the main determinant of NTD-associated morbidity, for some NTDs (including STH, schistosomiasis and onchocerciasis), the most severe clinical manifestations develop over many years of chronic or repeated infection. For these diseases, the association between infection and risk of long-term pathology is generally complex and poorly understood. The paper by Borlase *et al*. in this volume discusses the challenges for determining the relationship between cumulative pathogen exposure and morbidity at the individual and population levels, drawing on case studies for trachoma, schistosomiasis and foodborne trematode infections, and explores potential frameworks for explicitly incorporating long-term morbidity into NTD transmission models [28]. These frameworks are crucial for quantifying burden of disease, and the paper by Ledien and colleagues presents a modelling pipeline from serological surveys to morbi-mortality models for Chagas disease (an IDM) to quantify disease burden in time and space [29].

## 5. One Health

Recognizing that many of the NTDs are vector-borne, zoonotic or directly related to the interaction between the environment and human behaviour has led to a growing emphasis on the importance of animal-human-environmental interface by the NTD community. In particular, One Health is an approach that recognizes the connection between the health of people, animals and the environment and the need to build strong partnerships between these sectors. The tripartite collaboration between the WHO, the World Organisation of Animal Health and the United Nations Food and Agriculture Organization was formed to help countries to implement a One Health approach [30]. The paper by Díaz-Alvarado and co-authors discusses the importance of a One Health perspective for reaching the WHO elimination targets for schistosomiasis, highlighting that in Africa, by contrast with Asia, zoonotic components of schistosomiasis transmission and their implications for disease control have, until recently, been mostly ignored. They review recent epidemiological, clinical, molecular and modelling work across both Asia and Africa to emphasize the emerging risk raised by both wildlife reservoirs and viable hybridization between human and animal schistosomes [31]. The contribution by Qiu and collaborators proposes a multi-criteria qualitative approach to prioritize and characterize zoonoses for which surveillance in domestic animals is important to prevent human infections at a global scale [32].

## 6. Mathematical models

During the decade following the first WHO roadmap on NTDs [4], mathematical modelling (transmission dynamics, statistical and geostatistical frameworks) has played an increasing role in assisting the WHO, Ministries of Health policy-makers in endemic countries, and philanthropic organizations to assess how best to tackle NTDs and monitor programme progress, given the available intervention and diagnostic tools, as well as to evaluate the need for novel tools and support their development [33].

In an effort to synthesize and present the status of NTD models during the 2012–2020 period, two of the guest editors of this issue (M.G.B. and R.M.A.) edited two collections of comprehensive reviews of the then state-of-the-art mathematical models for NTDs as essential tools for control and elimination. These were published in 2015 and 2016 [34,35], and served as the basis on which policy-relevant models could be further developed, refined and improved.

During the WHO consultation process with the broad NTD community, initiated in 2019, NTD modellers were asked to provide insights, using improved models, into the revised targets for the 2021–2030 horizon, inform the technical feasibility of achieving them with current methods, ascertain the most salient risks, and identify the need for alternative and complementary strategies and novel tools. This endeavour, partly coordinated by the NTD Modelling Consortium (https://www.ntdmodelling.org/), and with contributions by other modelling consortia and groups gave rise to a collection of published pieces on dengue, rabies, trachoma, yaws, Chagas disease, gHAT, visceral leishmaniasis (VL), *T. solium* taeniasis, schistosomiasis, STH, LF, onchocerciasis and scabies (https://gatesopenresearch.org/collections/ntd), covering 13 of the 20 NTDs currently prioritized by the WHO [36].

In 2020, the COVID-19 pandemic severely disrupted health systems, MDA programmes, supply chains for NTD medicines and health products, and surveillance activities. These disruptions jeopardized support for prompt diagnosis, treatment and care, as well as provision of essential interventions such as vector control and veterinary public health [2]. NTD modellers were asked to evaluate the impact of such disruptions on seven NTDs (STH, *S. mansoni* schistosomiasis, LF, onchocerciasis, trachoma, VL and gHAT) and to identify mitigation strategies that would help bringing programmes against these NTDs back on track for reaching the proposed goals by 2030 [18,37,38].

Also in 2020, the NTD Modelling Consortium proposed the PRIME-NTD guidelines for Policy-Relevant Items to be included in Models for the Epidemiology of NTDs, based on the five principles of: (i) ensuring stakeholder engagement throughout the modelling process; (ii) providing complete model documentation and code; (iii) presenting complete description of the data used to fit/validate the model; (iv) quantifying and communicating uncertainty; and (v) reporting model outcomes in the form of testable hypotheses [39]. Some of the modelling papers in this collection have included the PRIME-NTD table to demonstrate that these principles have been followed (e.g. [9]).

For this issue, NTD modellers have contributed seven papers (50% of the contributions), including the potential of alternative treatment strategies for accelerating programmatic action towards attaining interruption of transmission [9]; the design of NTD impact surveys [12]; the usefulness of current and novel tools for diagnosis of helminthiases in areas ranging from low to high prevalence [17]; the feasibility and challenges posed by NTDs with long incubation periods and diagnostic delays [20]; the impact of human movement on reaching and sustaining elimination efforts [27]; the relationship between cumulative exposure to infection and development of severe morbidity [28], and the use of serological surveys and force-of-infection models linked to frameworks of disease progression for quantifying spatio-temporal patterns of disease burden accounting for uncertainty at all steps of the proposed pipeline [29]. The linkage between NTD models and data have certainly improved considerably over the past decade, but lessons learnt from model construction on what should be measured to better understand the impact of given control interventions on infection, transmission and morbidity have yet to filter through to the practical design of most monitoring and evaluation programmes.

Going forward, complex (stochastic) models should be fitted to baseline and longitudinal data (e.g. cross-sectional data across multiple time-points), and used to analyse the significance of spatial heterogeneity in both infection prevalence and intervention coverage. The model described by Collyer *et al.* [27] is the first, fully spatially dynamic NTD individual-based stochastic model of helminth transmission and control by MDA, with parameters estimated by fitting to a large spatially structured database recording infection prevalence and intensity at village level. However, much further work will be required in this area given a growing volume of data revealing much heterogeneity in MDA coverage at fine, village-level, spatial scales.

Further developments are also required in several other areas, including modelling prevalence data derived from diagnostic tools not reliant on microscopy-based detection of parasite stages (the classical outputs of most parasite transmission dynamics models); understanding how the performance of different diagnostics influences the interpretation of surveys for assessing control impact, post-treatment, post-validation and post-elimination surveillance; quantifying the epidemiological impact of adopting new drugs or new drug combinations and their cost-effectiveness, and reflecting how treatment adherence by individuals varies over a long sequence of MDA rounds.

## 7. Concluding remarks and future directions

This collection has shared some of the complex challenges faced as the NTD community works to achieve the targets set by the WHO to control, eliminate or eradicate its prioritized 20 NTDs by 2030 and beyond. As the three fundamental pillars of the new roadmap comprise accelerating programmatic action, intensifying cross-cutting approaches, and increasing country ownership, this volume has explored how these issues can only be addressed by international and multidisciplinary research teams working in tandem and in dialogue with national control programmes. Some papers have discussed the problem of long-term persistence of low prevalence for some NTDs, the need to account for spatial heterogeneity and to identify hot spots of infection prevalence, and the challenges for NTDs which slowly approach low incidence as transmission declines, as well as the need for alternative/complementary intervention strategies and more sensitive, diagnostic tools and techniques as we move closer to elimination.

Besides, most NTDs do not occur in isolation, but rather they are co-endemic with others, requiring integrated approaches to their diagnosis, treatment and control. The end is in sight for some NTDs, but issues related to the roles of surveillance and residual infection after MDA, verification/validation of elimination, and post-elimination surveillance, must be considered now to continue supporting, in the coming years, the investments made and the gains accrued [40].

Ten years have passed since the London Declaration on NTDs, and in 2022 the Kigali Declaration on NTDs, generated in consultation with stakeholders around the world, reaffirmed a global commitment to put individuals, communities, and countries at the centre of the NTD response [41]. Much progress has been achieved over this period in NTD control, especially for the PC infections where dug donations have had a major impact [7]. As the prevalence of infection falls for many of the NTDs, many and varied challenges emerge. These include: (i) sustaining donor interest, especially for drug supplies; (ii) stimulating research on more sensitive diagnostics and their development into field-friendly tools; (iii) understanding spatial heterogeneity in treatment coverage and how to achieve consistent levels of (increased) coverage to minimize the risk of pockets of infection acting to sustain transmission over various spatial scales; (iv) supporting the search for new drug options or drug combinations for particular infections or groups of infections; and last but not least (v) stimulating research activity in developing vaccines for NTDs that can go through clinical trials, since in the longer term they are likely to play a crucial role in achieving and protecting disease elimination.

## Data Availability

This article has no additional data.
